# Findings on Impact of Circadian Genes and Bipolar Disorder: A Bibliometric Analysis From 1996 to 2024

**DOI:** 10.1002/brb3.71274

**Published:** 2026-02-16

**Authors:** Zhuoer Ruan, Jie Zhu

**Affiliations:** ^1^ Department of Psychiatry The Second Affiliated Hospital and Yuying Children's Hospital of Wenzhou Medical University Wenzhou Zhejiang China

**Keywords:** bibliometrics, bipolar disorder, circadian genes

## Abstract

**Introduction:**

The disturbances of circadian genes are implicated in the pathophysiology of bipolar disorder (BD). This bibliometric analysis aims to explore global trends and hotspots in research on circadian genes and BD.

**Design:**

A bibliometric analysis.

**Methods:**

A systematic search was carried out on the Web of Science Core Collection (WoSCC) database to collect publications regarding circadian genes and BD. Subsequently, bibliometric analysis and visualization utilized VOSviewer (V 1.6.20), CiteSpace (V 6.3.R1), and the R package “Bibliometrix” (V 4.3.3).

**Results:**

The 400 articles involved 2405 authors from 1649 institutions, citing 18,840 sources in 166 journals. The number of publications has been consistently rising, with a 9.88% annual growth rate. The USA, Italy, and China dominated with the most articles. The most cited articles focused on the genetics of circadian disorders. The top institutions were the University of California system, the University of California, San Diego, and Université Paris. High‐impact authors included McClung Colleen A., McCarthy Michael J., and Benedetti Francesco. The top journals by H‐index were *Chronobiology International*, *Journal of Affective Disorders*, and *American Journal of Medical Genetics*. The keyword co‐occurrence analysis revealed focus on “depression,” “neurons,” “light therapy,” “genome wide association,” and “lithium.” The burst keywords highlighted the latest trends, including “sleep” and “brain,” with consistent emphasis on “risk.”

**Conclusion:**

This bibliometric analysis provides insights into the global trends and hotspots of research on circadian genes and BD. The hotspots are on symptoms, genetic associations, and treatment in BD, with frontiers exploring sleep patterns, brain functions, and risk factors.

## Graphical abstract

This study maps global research trends on circadian genes and bipolar disorder from 1996 to 2024 using bibliometric methods. Analysis of 400 articles reveals a steady growth in publications, with the USA, Italy, and China as leading contributors. Key research hotspots include depression, neuronal mechanisms, light therapy, genetic associations, and lithium treatment, with emerging focus on sleep, brain function, and risk factors. Major clusters represent clinical symptoms, molecular mechanisms, therapeutic strategies, genetic studies, and animal models. Findings provide insights for future research and clinical practice in psychiatric genetics.

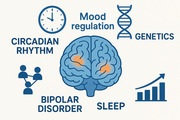

## 1 Introduction

Bipolar disorder (BD) is a complex and devastating psychiatric condition characterized by episodes of mania, hypomania, and depression, which may occur in various patterns, including periods of remission, rather than strictly alternating in an ABAB sequence (Carvalho et al. 2020). The global prevalence of BD is estimated at 1%–3% (Arnold et al. 2021; Tondo et al. 2017). The etiology of BD involves both genetic and environmental factors, including alterations in neurotransmitter systems (such as serotonin, dopamine, and glutamate), dysregulation in brain regions responsible for emotion and cognition, and exposure to environmental stressors (Barnett and Smoller 2009; Harrison et al. 2018). Current treatment strategies for BD rely primarily on pharmacological interventions, psychotherapeutic approaches, and lifestyle modifications (Vieta et al. 2018; Solé et al. 2017). However, due to the incomplete understanding of BD's underlying mechanisms, ongoing research seeks more effective and individualized therapies.

A growing body of evidence supports the involvement of circadian rhythms in the pathophysiology of BD (Chung et al. 2023). Circadian genes—also referred to as clock genes—are a set of highly conserved genes that regulate the endogenous 24‐h biological cycles, influencing sleep‐wake patterns, hormonal secretion, and mood regulation. The “core clock genes” are a subgroup of circadian genes, including CLOCK, BMAL1 (ARNTL1), PER1, PER2, PER3, CRY1, and CRY2, which form transcriptional‐translational feedback loops to generate and maintain circadian rhythms (Chung et al. 2023; Oliveira et al. 2018). Disruptions or altered expressions of these genes have been observed in BD patients (Oliveira et al. 2018). Seminal studies, such as those by McClung et al. and Takahashi et al., have established the molecular underpinnings of the circadian hypothesis of BD, demonstrating that mutations or dysregulation of core clock genes can lead to abnormal mood regulation and behavioral phenotypes resembling BD in animal models.

Courtin et al. (2023) proposed a chronobiological model of BD, emphasizing the importance of imbalances in core clock gene expression between BD patients and healthy controls, as validated by RNA sequencing and RT‐qPCR. Variants in circadian genes, such as TIMELESS and RORA, have been associated with altered circadian phenotypes and increased susceptibility to BD (Etain et al. 2014). Chronotype, particularly evening preference, has been linked to worse outcomes in BD (Zou et al. 2022). Furthermore, disruptions in the Period (Per) and Cryptochrome (Cry) gene families impair sleep‐wake cycles and mood regulation (Faltraco et al. 2022; C. Zhang et al. 2022). Notably, RORA functions as a key regulator of the circadian clock, and its abnormal variants contribute to eveningness, languidness, and rigid circadian types (Maher et al. 2015). These findings underscore the importance of further investigating the role of circadian genes in BD to improve our understanding and treatment of the disorder.

Given the burgeoning interest in the intersection of circadian genes and BD, a focused bibliometric analysis is warranted to elucidate global research trends (Y. Zhang, Chen, et al. 2023). Previous bibliometric studies, such as those by He et al. (2023) and Zakaria et al. (2023), have demonstrated significant growth and evolving hotspots in the fields of insomnia, circadian rhythm, and genetic research related to BD. However, a dedicated bibliometric analysis specifically targeting circadian genes in BD remains lacking.

Therefore, the present study aims to fill this gap by systematically analyzing publications related to circadian genes and BD from 1996 to 2024. By using advanced bibliometric tools and methodologies, we seek to map global trends, identify key research hotspots, and highlight areas for future exploration.

## 2 Materials and Methods

### 2.1 Data Source and Literature Search Strategy

A thorough literature review was conducted to identify publications related to circadian genes and BD. The search was performed in the Web of Science Core Collection (WoSCC), which is a widely used interdisciplinary citation database that covers a broad range of scholarly journals and provides comprehensive bibliometric data (Tao et al. 2023). The WoSCC includes records from Science Citation Index Expanded (SCI‐EXPANDED), Social Sciences Citation Index (SSCI), and Arts & Humanities Citation Index (A&HCI). Only articles and reviews published in English were considered for inclusion, as WoSCC allows filtering by language and document type (Liu et al. 2024). The search formula was (TS = (“bipolar disorder*” OR “bipolar mood disorder*” OR “bipolar affective psychos$s” OR “manic‐depressive psychos$s” OR “manic depressive psychos$s” OR “bipolar depression*” OR “manic disorder*” OR “manic depression*” OR “bipolar affective disorder*” OR “bipolar depressive disorder*” OR “bipolar spectrum disorder*” OR “biphasic disorder*”)) AND TS = (“circadian gene*” OR “clock controlled gene*” OR “clock gene*” OR “circadian clock gene*” OR ((CRY OR CRY1 OR CRY2 OR PER1 OR PER2 OR PER3 OR NR1D1 OR NR1D2 OR ROR OR RORA OR RORB OR RORC OR DEC OR DEC1 OR DEC2 OR CSNK1 OR CSNK1D OR CSNK1E OR ARNTL1 OR ARNTL2 OR BMAL1 OR NPAS2 OR CLOCK) And gene*)). The search was conducted on December 11, 2024. No restrictions were placed on publication year at the time of search. Only English‐language articles and reviews were included.

**FIGURE 1:**
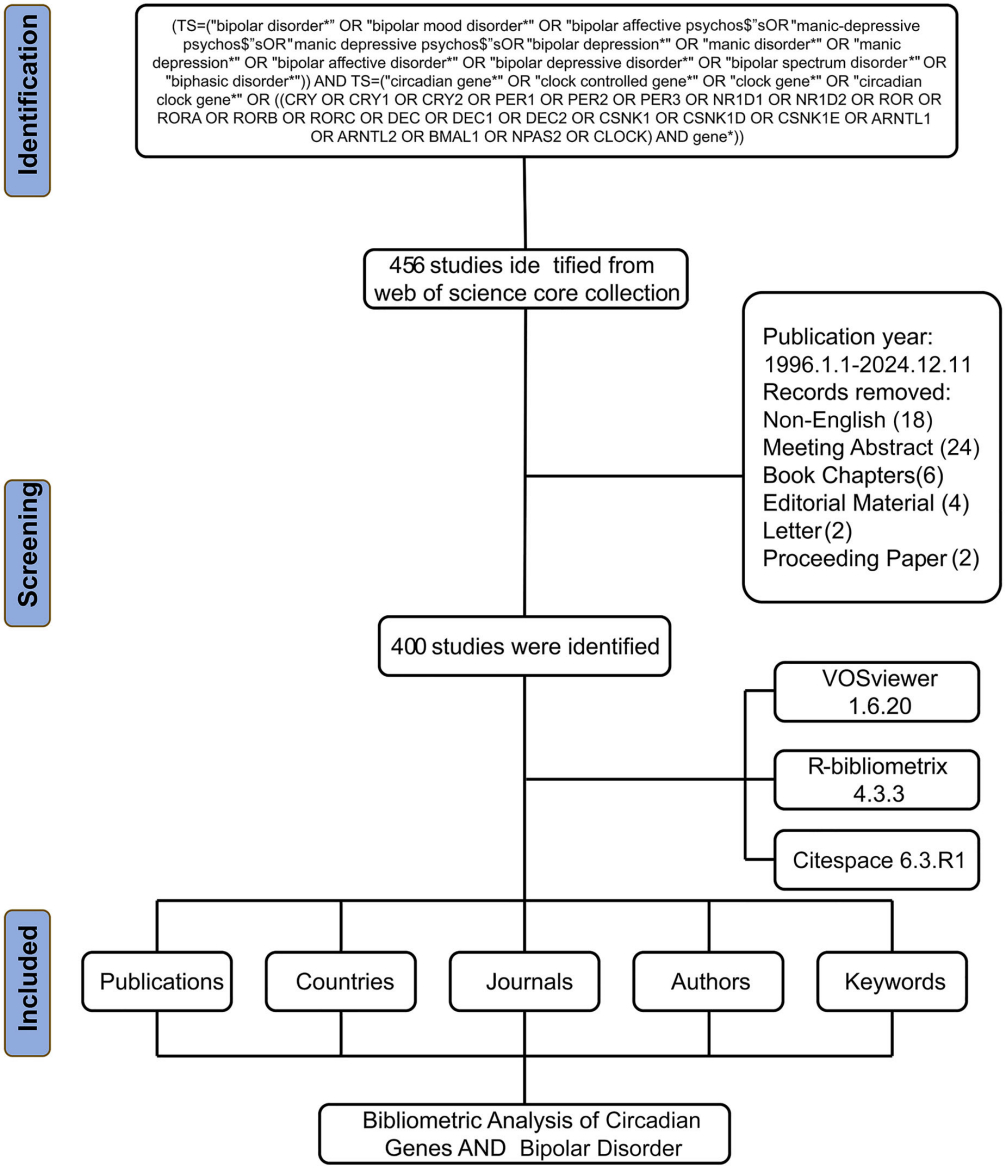
Flow diagram of the bibliographic retrieval process in the field of circadian genes and bipolar disorder.

To ensure transparency and reproducibility, the complete search strategy, including all search terms and parameters, is detailed above. The search results were independently reviewed by two researchers to confirm eligibility. Discrepancies were resolved by discussion and consensus. Duplicates and non‐relevant records (e.g., meeting abstracts, book chapters, editorials, letters, and proceedings) were excluded during the screening process. A PRISMA flow diagram (Figure 1) was constructed to document the selection process. The following bibliometric indicators were extracted from eligible records: publication count, citation data, article titles, country/region of origin, institutional affiliations, journal information, author details, and keywords.

### 2.2 Statistical Analysis

For data processing, all retrieved records were exported from WoSCC in plain text format and imported into Microsoft Excel for initial organization and deduplication. Subsequently, VOSviewer (V 1.6.20), CiteSpace (V 6.3.R1), and the R package “Bibliometrix” (V 4.3.3) were used for bibliometric analysis and visualization.

VOSviewer generated network visualizations mapping co‐occurrences between countries, institutions, authors, and keywords. Node size represented frequency, color indicated clusters or publication year, and line thickness showed connection strength (Jia and Mustafa 2022). CiteSpace analyzed research trends from 1996 to 2024 using time‐zone visualizations, with keywords as nodes in annual segments to track thematic evolution, highlighting five key terms per period (C. Zhang, Zhang, et al. 2023). The R package “Bibliometrix” was used to calculate bibliometric indicators, including the H‐index, G‐index, and M‐index, for notable authors and institutions. The H‐index measured productivity and impact (from WoSCC data), the G‐index weighted highly‐cited papers, and the M‐index adjusted the H‐index for the length of an academic career (Bertoli‐Barsotti and Lando 2017; Garg et al. 2022; Mondal and Mondal 2022). To ensure the robustness and reliability of the results, all analyses were independently performed by two researchers. Any discrepancies were resolved through discussion. To assess journal influence, metrics from Journal Citation Reports (JCR), including the 2023 impact factor (IF) and quartile ranking (Q1–Q4), were collected. The IF reflects the average number of citations per article in a specific year, with quartile ranking dividing journals into four tiers (Q1 representing the top 25%).

## 3 Results

### 3.1 Overview of Publications

**FIGURE 2:**
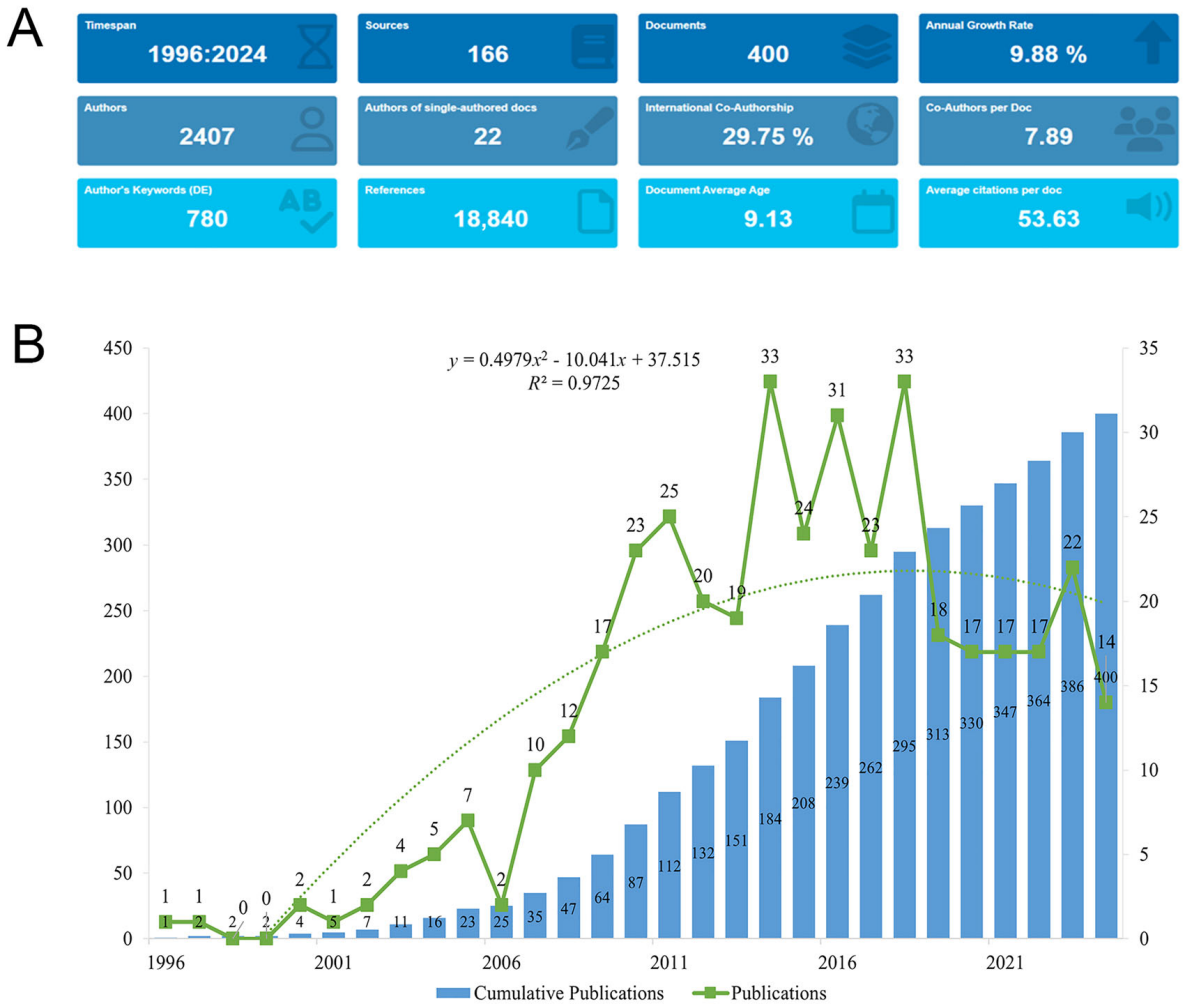
Analysis of the general information in the field of circadian genes and bipolar disorder. (A) A summary of quantitative analysis of the publications in the field of circadian genes and bipolar disorder. (B) Annual output of research from 1996 to 2024.

Our study identified and included 400 eligible English articles between 1996 and 2024 pertaining to circadian genes and BD, excluding 18 non‐English publications, 24 meeting abstracts, 6 book chapters, 4 editorial materials, 2 letters, and 2 proceeding papers (Figure 1). In 1996, only two articles were published in this field, indicating minimal initial research activity. Over subsequent years, publication output increased steadily. These publications involved a diverse group of 2405 authors, who were affiliated with 1649 institutions globally. Across 166 journals, these articles cited a substantial total of 18,840 sources (Figure 2A).

Between 1996 and 2024, the number of articles in this field consistently rose. By 2024, the total number has surged to 400. The annual growth rate of publications stood at 9.88%, with a fitting equation of *y* = 0.4979*x* ^2^ − 10.041*x* + 37.515 (*R* ^2^ = 0.9725). The annual number of publications peaked at 33 articles in both 2014 and 2018, with a slight decline in recent years (Figure 2B).

The top three most cited articles in this field were “The genetics of mammalian circadian order and disorder: implications for physiology and disease” published in the *Nature Reviews Genetics* (IF = 39.1) in 2008, “Mania‐like behavior induced by disruption of CLOCK” published in the *Proceedings of the National Academy of Sciences of the United States of America* (IF = 9.4) in 2007, and “Circadian genes, rhythms and the biology of mood disorders” published in the *Pharmacology & Therapeutics* (IF = 12) in 2007, with 1182, 595, and 495 citations, respectively (Table S1).

### 3.2 Contributions and Collaborative Networks of Journals

Among the high‐impact journals ranked by H‐index, *Chronobiology International* (IF 2023 = 2.2, Q3) and *Journal of Affective Disorders* (IF 2023 = 4.9, Q1) were tied for first place with an H‐index of 15 for both. The former contributed to 20 publications (ranking second) with 654 citations, while the latter contributed to 23 publications (ranking first) with 823 citations. In third place was *American Journal of Medical Genetics* (IF 2023 = 1.6, Q3), with an H‐index of 12, 13 publications, and 702 citations. Of the top 20 high‐impact journals, *Lancet Psychiatry* achieved the highest IF in 2023 (IF 2023 = 30.8, Q1) (Table S2).

**FIGURE 3:**
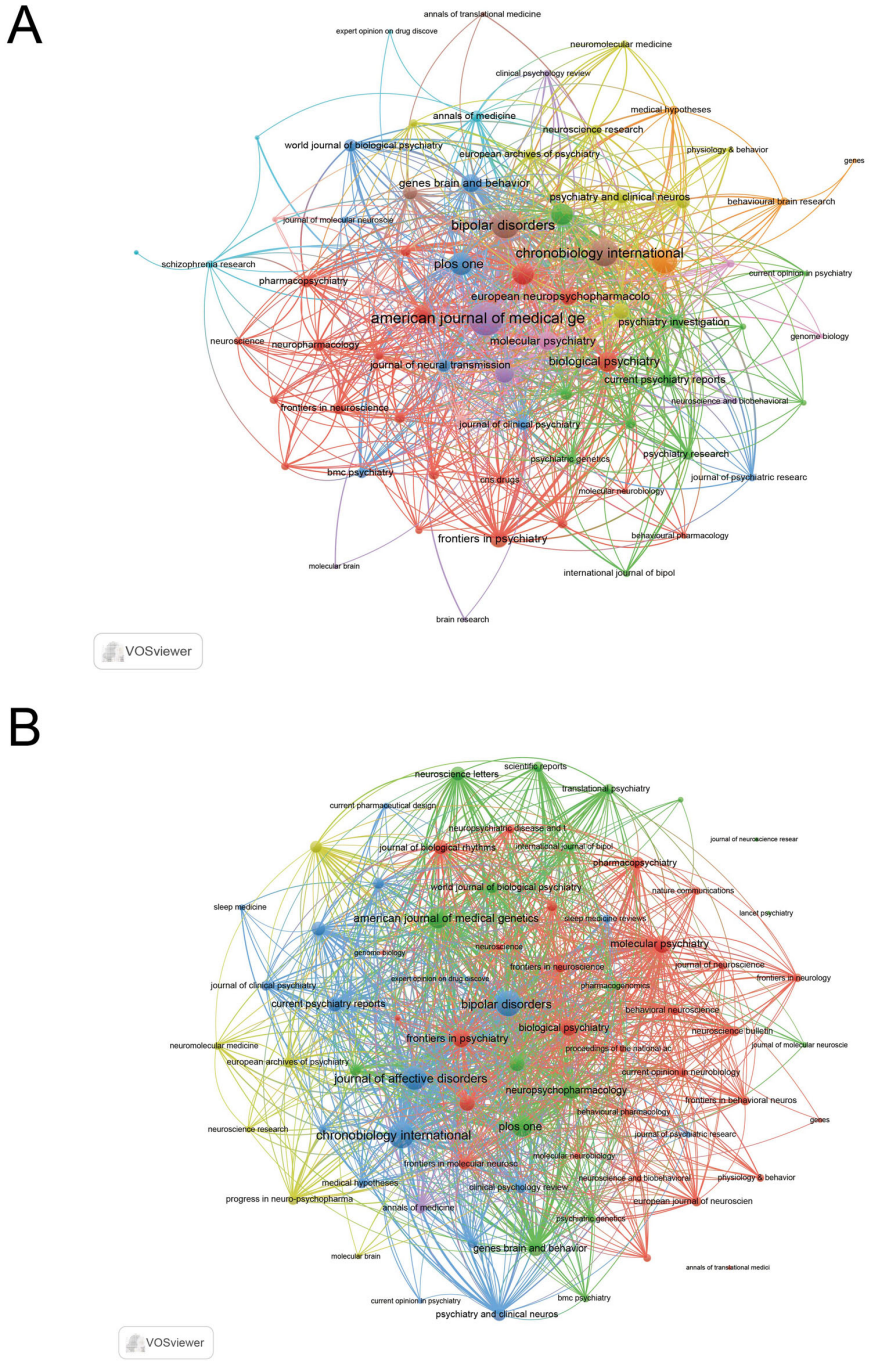
Analysis of journals in the field of circadian genes and bipolar disorder. (A) The co‐occurrence networks of journals. (B) The coupling networks of journals.

The co‐occurrence network analyzed journal citations in articles, revealing thematic coherence through significant interactions. In this network, journals are connected when they are cited together in the same articles, indicating shared research interests or thematic overlap among the cited literature. Stronger connections suggest that certain journals frequently co‐appear in the reference lists, highlighting their central role in the field. Among the 166 journals with at least two occurrences, the top three were *American Journal of Medical Genetics Part B‐Neuropsychiatric Genetics* (strength = 384), *Bipolar Disorders* (strength = 256), and *Chronobiology International* (strength = 252) (Figure 3A).

The coupling network assessed interconnectedness by examining common references among articles. High strength in this network indicates substantial overlap in shared references, reflecting a strong intellectual foundation. Among the 166 journals with at least two couplings, Chronobiology International (strength = 9144), *Bipolar Disorders* (strength = 8586), and *Journal of Affective Disorders* (strength = 7738) stood out as the top three (Figure 3B).

### 3.3 Distribution and Collaborative Networks of Countries

**FIGURE 4:**
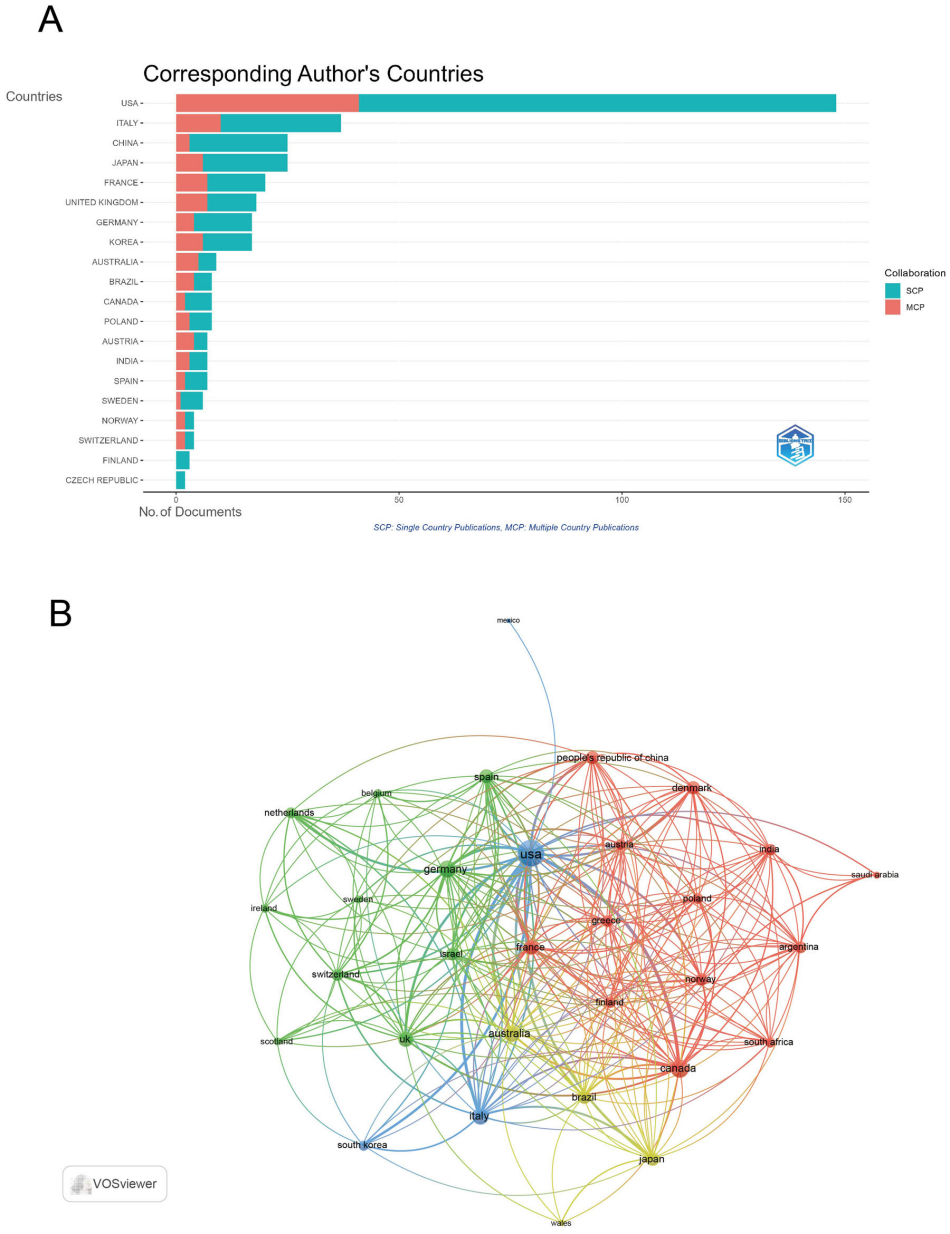
Analysis of countries in the field of circadian genes and bipolar disorder. (A) Distribution of responding authors’ publications by country. (B) Visualization map depicting collaboration among different countries.

The USA significantly outpaced other countries in article volume, accounting for 37.0% of the total with 148 articles. It also topped the list in multiple country publications (MCP = 41) and received the highest number of total citations (TC = 11,381). Italy followed in second place in article volume (*n* = 37, 9.3%), MCP = 10, and TC = 1998. China came in third in terms of article volume, with 25 articles (6.3%) (Figure 4A, Table S3).

The USA not only published the greatest number of articles but also shone in international collaborations. Among the 48 countries engaged in international collaborations with at least three articles, the USA (strength = 137) led with the highest number of collaborations, closely followed by Canada (strength = 61) and Germany (strength = 55) (Figure 4B).

### 3.4 Distribution and Collaborative Networks of Institutions

**FIGURE 5:**
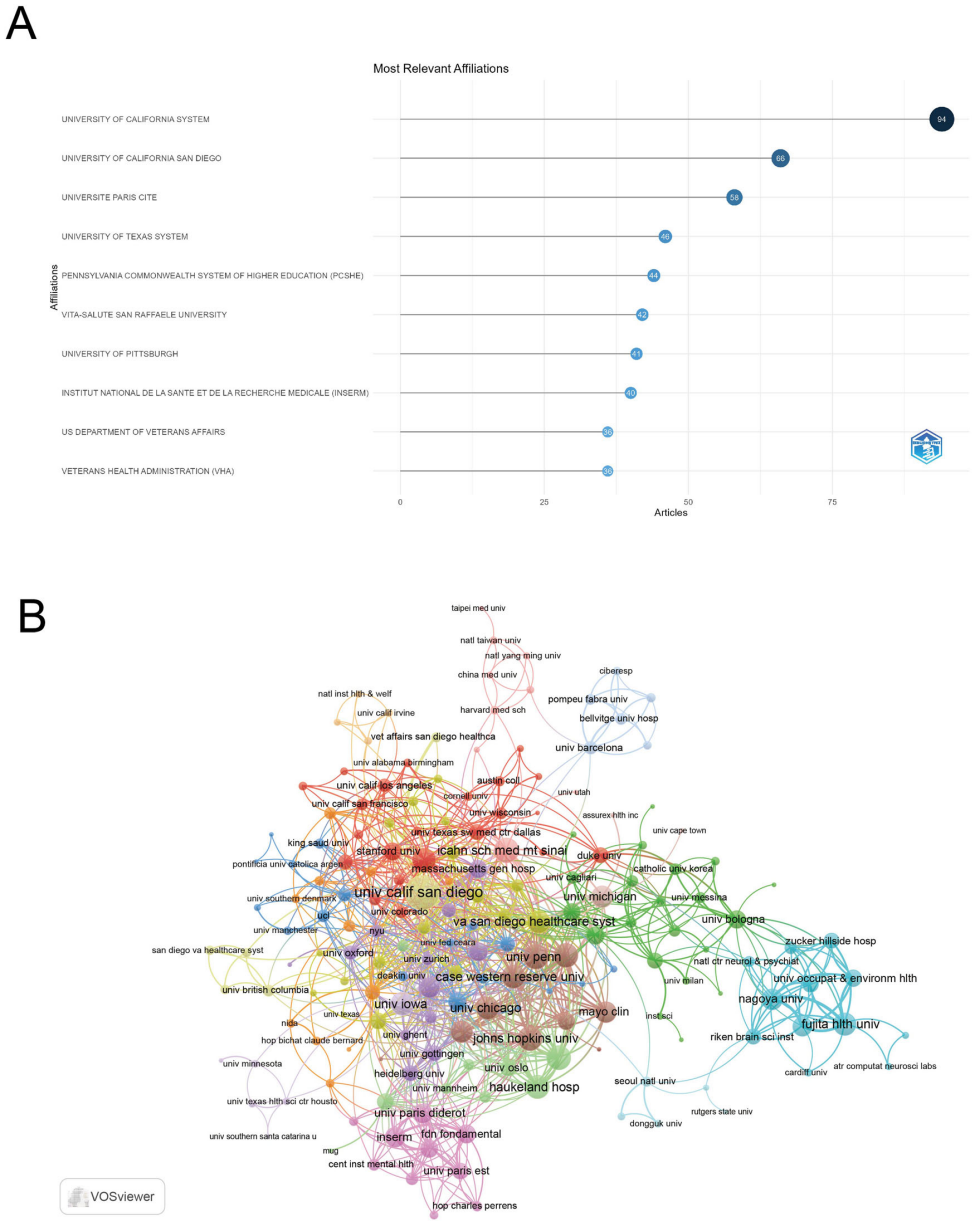
Analysis of institutions in the field of circadian genes and bipolar disorder. (A) Top 10 institutions ranked by article count. (B) Visualization map depicting collaboration among different institutions.

Institutions are ranked by article numbers and listed in Figure 5A. The top three were respectively the University of California System (*n* = 94) in the USA, the University of California, San Diego (*n* = 66) in the USA and Université Paris Cité (*n* = 58) in France.

A total of 738 institutions participated in collaborations, each contributing at least two articles. Multiple major research clusters have formed among these institutions, indicated by different colors. Among them, the University of California, San Diego led with the highest number of collaborations (strength = 115), closely followed by the University of Pennsylvania (strength = 59) and the Icahn School of Medicine at Mount Sinai (strength = 58), all located in the USA. These three institutions belonged to the yellow, brown, and pink clusters, respectively (Figure 5B).

### 3.5 Authors and Co‐Authors

The authors were ranked by their H‐index, with McClung Colleen A. leading the high‐impact authors with an H‐index of 19. This author also contributed to the most total publications (TP = 21) and achieved the most citations (TC = 2422). Michael J. McCarthy followed with an H‐index of 14, 16 publications (ranking second), and 831 citations. In third place was Benedetti Francesco with an H‐index of 11, 12 publications (ranking third), and 513 citations (Table S4).

**FIGURE 6:**
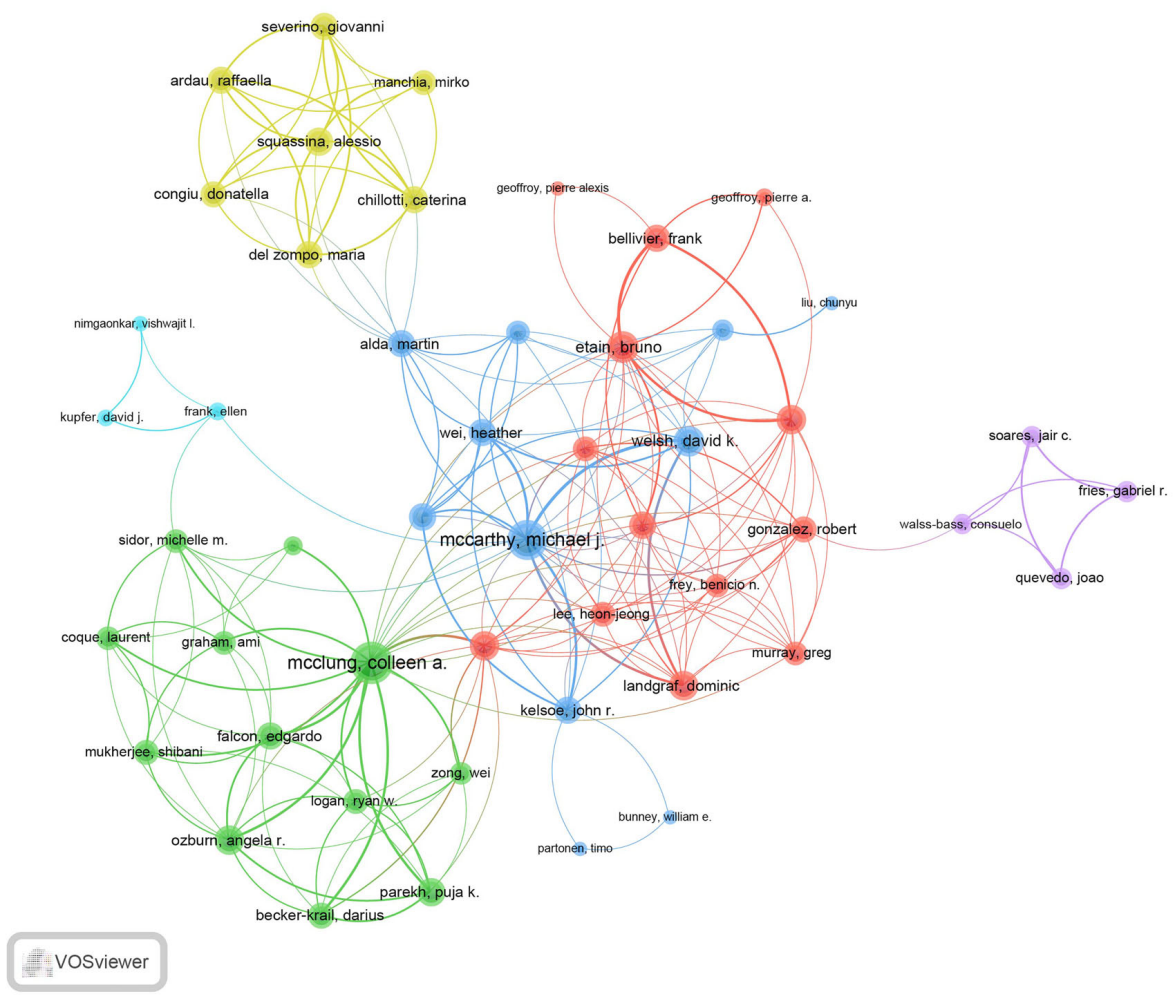
The visualization map depicting collaboration among authors in the field of circadian genes and bipolar disorder.

Out of the 2139 authors engaged in collaborations with a minimum of three articles, Iwata, Nakao topped the list with the highest number of collaborations (strength = 117), closely trailed by Kishi, Taro (strength = 116) and Kitajima, Tsuyoshi (strength = 109) (Figure 6).

### 3.6 Keyword Co‐Existence Network and Burst Keywords

**FIGURE 7:**
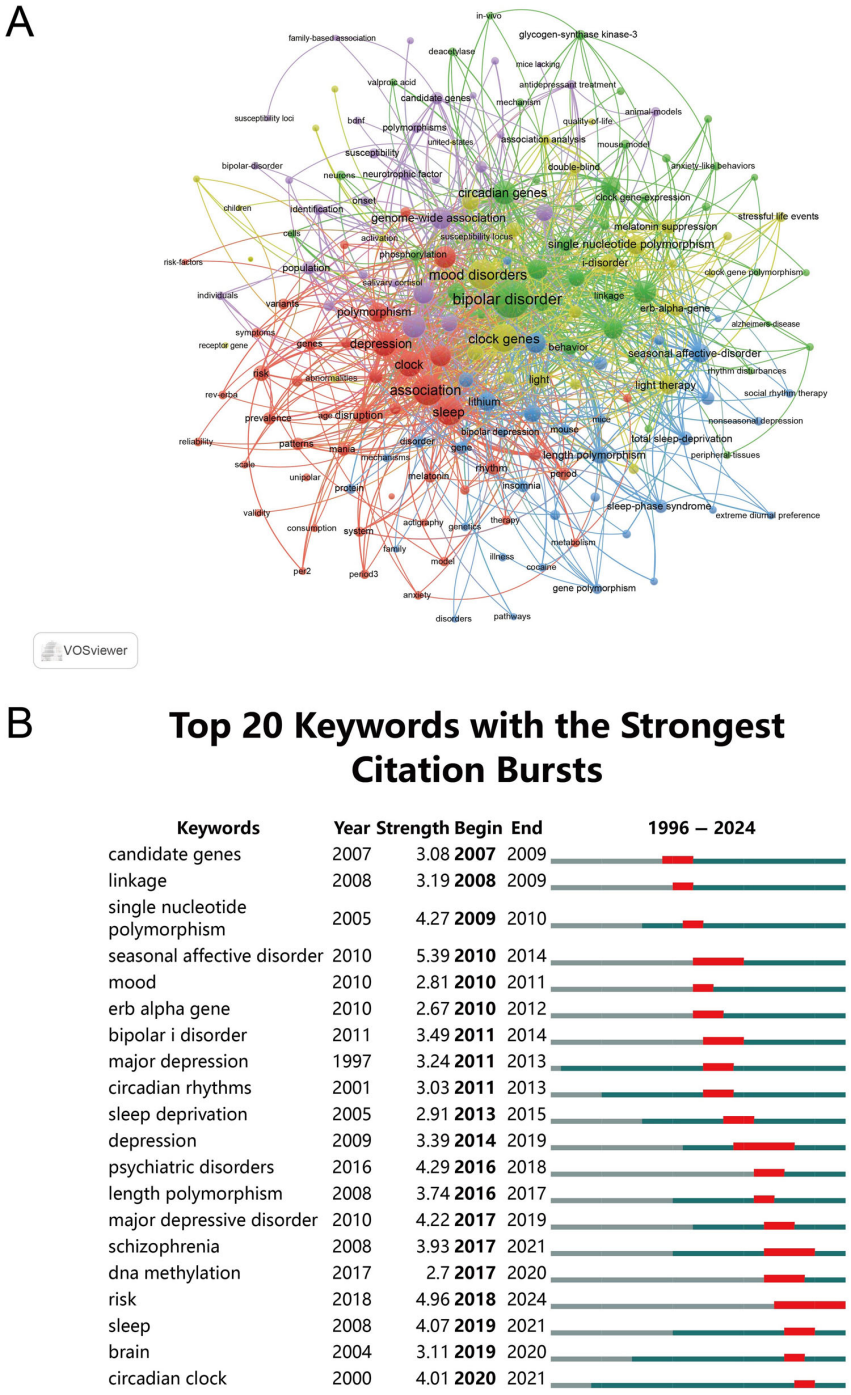
Analysis of keywords in the field of circadian genes and bipolar disorder. (A) Visual analysis of keyword co‐occurrence network. (B) Top 20 keywords with the strongest citation bursts.

In total, 1390 keywords with a minimum of four occurrences were identified. Cluster 1 (red) focuses on the manifestations of BD, with “depression,” “symptoms,” and “period” appearing frequently. Cluster 2 (green) centers around the molecular and cellular mechanisms related to the disorder, as indicated by prominent keywords like “neurons,” “phosphorylation,” and “glycogen synthase kinase‐3.” Cluster 3 (yellow) is concerned with therapeutic interventions and molecular targets, with prominent keywords including “light therapy,” “Rev‐Erb alpha,” and “melatonin suppression.” Cluster 4 (purple) emphasizes large‐scale genetic studies and related factors, including “genome wide association,” “population,” and “neurotrophic factor.” Cluster 5 (blue) is centered around therapeutic interventions, related disorders, and animal models, including “lithium,” “seasonal affective disorder,” and “mouse” (Figure 7A).

The evolution of hotspots in this field from 1996 to 2024 is evident from the burst keywords depicted in Figure 7B. Prior to 2013, the research focus was centered around topics such as “single nucleotide polymorphism” (strength = 4.27) and “sleep deprivation” (strength = 2.91). Between 2014 and 2016, key burst terms encompassed “length polymorphism” (strength = 3.74) and “DNA methylation” (strength = 2.7). Since 2017, there has been a significant increase in research on “sleep” (strength = 4.07) and “brain” (strength = 3.11). In addition, the prominence of “risk” (strength = 4.96) has persisted consistently until 2024.

## 4 Discussion

This bibliometric analysis evaluated global trends, research hotspots, and evolving directions in circadian gene and BD research from 1996 to 2024, identifying 400 eligible English articles. Our findings reveal a steady increase in publications over the years, with notable changes in the focus and methodology of studies during this period.

The annual publication trend showed a marked rise, especially after 2010. While our original analysis attributed this growth to advances in genetic sequencing technologies, it is more accurate to note that much of the genetic research during this era utilized PCR‐based methods and high‐throughput chip‐based genotyping platforms for genome‐wide association studies (GWAS), rather than next‐generation sequencing. The increased accessibility of these tools, along with broader research funding and international collaboration, facilitated the identification of genetic variants associated with BD. In addition, the development and widespread use of bioluminescent reporter genes, such as Per2‐luciferase (Per2‐luc) mice and cell lines, significantly advanced the study of circadian rhythms in living cells and animal models, enabling real‐time monitoring of molecular clock dynamics (Takaesu 2018).

A major milestone for the field was the awarding of the 2017 Nobel Prize in Physiology or Medicine to Jeffrey C. Hall, Michael Rosbash, and Michael W. Young for their discoveries of molecular mechanisms controlling circadian rhythms (Callaway and Ledford 2017; Van Laake et al. 2018). This global recognition spurred a temporary surge of interest and publications in circadian biology, including its relevance to BD. Interestingly, our data show that publication activity in this area peaked around this period but has declined slightly in recent years, a trend also observed in other bibliometric analyses of circadian rhythm research.

The most cited articles in this field focused on the genetics of circadian disorders and their implications for BD. Takahashi et al. (2008) provided a molecular understanding of circadian biology's influence on physiological processes related to human diseases, elucidating clock genes' molecular mechanisms in disease. Roybal et al. (2007) demonstrated CLOCK gene's influence on manic‐like behaviors via dopamine regulation. Colleen linked circadian rhythms to mood disorders, including the roles of clock genes and their impact on brain regions and neurotransmitter systems involved in mood regulation (McClung 2007). Notably, Michael J. McCarthy, highlighted in our abstract as a leading contributor, has been instrumental in advancing cellular models of BD. McCarthy's research pioneered the use of patient‐derived fibroblasts and induced pluripotent stem cell (iPSC)‐derived neurons to investigate circadian rhythm abnormalities and lithium response in BD. Recent work from his group has shown that circadian rhythm disruptions in BD patient‐derived neurons predict clinical response to lithium, providing a potential platform for personalized medicine (Mishra et al. 2023, 2021). These cellular models have also enabled mechanistic studies of core clock gene expression, post‐translational modifications, and pharmacological interventions, bridging molecular circadian biology and clinical practice.

The USA and its institutions dominated the research landscape, likely due to a combination of high BD prevalence, substantial biomedical funding, and advanced infrastructure (Clemente et al. 2015). Italy and China also showed strong productivity. While international collaboration was robust, enhanced partnerships could further accelerate discoveries. Influential authors and high‐impact journals in chronobiology and psychiatry have driven progress in research at the intersection of circadian biology and BD.

Analysis of research clusters provides valuable insights into the thematic organization and evolution of the field. As demonstrated in prior bibliometric studies (He et al. 2023; Y. Zhang, Chen, et al. 2023), cluster analysis allows for the identification of major research domains and emerging trends. In this study, clusters were identified based on keyword co‐occurrence networks, facilitating a nuanced understanding of research foci within circadian genes and BD. Such cluster‐based discussion has been used in other bibliometric reviews to provide structure to the interpretation of complex research landscapes (Zakaria et al. 2023).

### 4.1 Cluster 1 (Red): Manifestations of BD

The red cluster describes the clinical symptoms of BD, including depression and mania, and their patterns of occurrence (Tondo et al. 2017). Research in this cluster has informed clinical phenotyping and the development of diagnostic criteria.

### 4.2 Cluster 2 (Green): Molecular Mechanisms of BD

The green cluster revealed BD's molecular underpinnings, highlighting keywords like “neurons,” “phosphorylation,” and “glycogen synthase kinase‐3” (GSK‐3). Neuronal signal disturbances and synaptic dysfunction are characteristic features of BD, contributing to mood and cognitive symptoms (Mishra et al. 2021). Phosphorylation, as a post‐translational modification, is critically involved in regulating clock gene activity. For example, Li et al. (2019) demonstrated that disruptions in CAMK2A‐mediated phosphorylation contribute to BD pathophysiology, while Yang et al. (2008) reported that reduced GSK3beta phosphorylation is a key feature in BD. FGSK‐3, a circadian‐regulated kinase, modulates synaptic plasticity and mood regulation, making it a promising therapeutic target (Mohawk et al. 2009; Benedetti et al. 2004).

### 4.3 Cluster 3 (Yellow): Therapeutic Interventions and Molecular Targets

The yellow cluster is characterized by research on interventions such as light therapy, Rev‐Erb alpha, and melatonin suppression. Light therapy has been shown to reduce depressive symptoms in BD without increasing the risk of manic switches (Lam et al. 2019). The Rev‐Erb alpha gene encodes a nuclear receptor involved in circadian regulation, with genetic variants linked to BD risk (Campos‐de‐Sousa et al. 2010). Melatonin and its receptors, particularly MT1, are also implicated in circadian dysfunction in BD, and recent studies have explored their potential as therapeutic targets (Tassan Mazzocco et al. 2023).

### 4.4 Cluster 4 (Purple): Large‐Scale Genetic Studies

The purple cluster encompasses GWAS, population studies, and research on neurotrophic factors. GWAS have identified associations between circadian gene variants (e.g., CRY1, NPAS2, VIPR2, CLOCK, VIP) and mood disorders, supporting the hypothesis that different clock genes may influence the polarity and course of illness (Soria et al. 2010). Neurotrophic factors, which promote neuronal growth and survival, further underscore the importance of neuroplasticity in BD (Le‐Niculescu et al. 2021).

### 4.5 Cluster 5: Therapeutic Interventions, Related Disorders, and Animal Models

The blue cluster integrates research on lithium, seasonal affective disorder, and animal models. Lithium, the mainstay mood stabilizer for BD, has been shown to modulate clock gene expression, and its therapeutic mechanism may be mediated by interactions with circadian genes such as PER1, PER3, and RORA (Geoffroy et al. 2017, 2016; Mishra et al. 2023). Seasonal affective disorder shares similar circadian disruptions with BD, and animal models, including Per2‐luc transgenic mice, have been crucial for dissecting the molecular basis of these disorders.

The evolution of research hotspots, as shown by burst keyword analysis, highlights the shifting focus of the field. Before 2013, studies primarily explored the impact of sleep deprivation on BD symptoms and circadian gene regulation (Bunney and Bunney 2013). Between 2014 and 2016, epigenetic modifications, such as length polymorphisms in Per3 and DNA methylation of melatonin receptor genes, were recognized as potential contributors to BD risk and diagnostic markers (Pandi‐Perumal et al. 2014; Lesicka et al. 2023). Since 2017, research has increasingly focused on sleep disturbances, brain function, and risk factors, reflecting a more holistic view of BD pathophysiology and progression (Calabrò et al. 2018; Xu et al. 2022).

Despite these advances, several knowledge gaps and challenges remain. Many studies are cross‐sectional or based on small sample sizes, limiting causal inference and generalizability. The integration of multi‐omics data (genomics, epigenomics, and transcriptomics) is still in its infancy, and the translation of basic circadian findings into clinical practice faces obstacles, including variability in individual response and the complexity of mood disorder phenotypes.

Emerging research areas offer promising avenues for future investigation. Digital therapeutics and mobile health (mHealth) applications are increasingly being used to monitor circadian rhythms, sleep patterns, and mood in real‐time, providing new opportunities for personalized intervention and early detection of mood episodes. These technologies have the potential to bridge gaps between laboratory research and clinical care by integrating circadian biomarkers into BD management, thus facilitating more precise and dynamic treatment strategies.

### 4.6 Strengths and Limitations

This study conducted an exhaustive review of 400 eligible English articles on circadian rhythm genes and BD spanning from 1996 to 2024, ensuring a comprehensive understanding of the field. Furthermore, the study employed various metrics such as the H‐index, citation counts, and collaboration strengths to provide a nuanced view of the impact and influence of researchers, institutions, and journals. In addition, the use of co‐occurrence and coupling networks to analyze journal citations and interconnectivity among articles provided insights into the thematic consistency and intellectual foundation of the field.

Several strengths of this study should be highlighted. First, the application of robust bibliometric tools and multiple indicators (e.g., H‐index, G‐index, collaboration networks) strengthens the reliability of the findings. Second, the inclusion of a 28‐year period captures both historical and contemporary trends. Third, the use of network analysis offers a more granular view of collaborative patterns and thematic development.

Despite these strengths, several limitations should be noted. First, the bibliometric analysis relied solely on the WoSCC database. While WoSCC is a widely used and reputable source that ensures high‐quality and consistent indexing (Tao et al. 2023), depending on a single database may result in the omission of relevant articles indexed only in other databases (e.g., Scopus, PubMed, Embase). This limitation is recognized in recent bibliometric literature, where single‐database approaches have been justified for data quality and reproducibility, but are acknowledged as potentially less comprehensive (Y. Zhang, Chen, et al. 2023). Therefore, future studies should consider incorporating multiple databases to maximize coverage and minimize publication bias.

Second, the analysis was limited to English‐language articles, which may have excluded relevant research published in other languages. Third, bibliometric indicators such as citation counts and H‐indices may not fully capture the quality, impact, or novelty of individual studies. Finally, keyword analysis may have been influenced by the specific search terms used and the indexing practices of WoSCC.

## 5 Conclusion

This bibliometric analysis assessed the global trends and hotspots of circadian genes and BD from 1996 to 2024. The research hotspots centered around depression, neuronal mechanisms, light therapy, genetic associations, and lithium treatment in circadian genes and BD, while the research frontiers focused on sleep patterns, brain functions, and risk factors. The findings of this study contribute to a better understanding of the global research landscape in this field and provide valuable insights for future research directions.

## Author Contributions

Conception and design: Jie Zhu. Administrative support: Jie Zhu. Data analysis and interpretation: Zhuoer Ruan. Manuscript writing: Zhuoer Ruan and Jie Zhu. Final approval of manuscript: Zhuoer Ruan and Jie Zhu.

## Funding

The authors have nothing to report.

## Ethics Statement

The authors have nothing to report.

## Consent

The authors have nothing to report.

## Conflicts of Interest

The authors declare no conflicts of interest.

## Supporting information




**Supplementary Tables**: brb371274‐sup‐0001‐TableS1‐S4.docx

## Data Availability

All data generated or analyzed during this study are included in this published article.
