# Supplementary material for: Findings on Impact of Circadian Genes and Bipolar Disorder: A Bibliometric Analysis From 1996 to 2024
Source: Brain Behav. 2026 Feb 16;16(2):e71274. doi: 10.1002/brb3.71274 (PMC12910130; doi:10.1002/brb3.71274)
Supplement: Supplementary file 1 — Supplementary Tables: brb371274‐sup‐0001‐TableS1‐S4.docx [file BRB3-16-e71274-s001.docx]

**Table S1.** Top 50 articles with the highest cited counts.

| **Paper** | **DOI** | **TC** | **TC per year** | **Normalized TC** |
| --- | --- | --- | --- | --- |
| TAKAHASHI JS, 2008, NAT REV GENET | 10.1038/nrg2430 | 1182 | 69.53 | 6.06 |
| ROYBAL K, 2007, P NATL ACAD SCI USA | 10.1073/pnas.0609625104 | 595 | 33.06 | 3.19 |
| MCCLUNG CA, 2007, PHARMACOL THERAPEUT | 10.1016/j.pharmthera.2007.02.003 | 495 | 27.5 | 2.66 |
| YIN L, 2006, SCIENCE | 10.1126/science.1121613 | 428 | 22.53 | 1.32 |
| HARVEY AG, 2011, CLIN PSYCHOL REV | 10.1016/j.cpr.2010.04.003 | 406 | 29 | 5.75 |
| MCCLUNG CA, 2013, BIOL PSYCHIAT | 10.1016/j.biopsych.2013.02.019 | 345 | 28.75 | 5.54 |
| LASKY-SU J, 2008, AM J MED GENET B | 10.1002/ajmg.b.30867 | 303 | 17.82 | 1.55 |
| SORIA V, 2010, NEUROPSYCHOPHARMACOL | 10.1038/npp.2009.230 | 266 | 17.73 | 3.62 |
| BENEDETTI F, 2003, AM J MED GENET B | 10.1002/ajmg.b.20038 | 254 | 11.55 | 1.96 |
| ASTON C, 2004, J NEUROSCI RES | 10.1002/jnr.20208 | 243 | 11.57 | 1.72 |
| CHUNG S, 2014, CELL | 10.1016/j.cell.2014.03.039 | 228 | 20.73 | 4.89 |
| KATO T, 2007, PSYCHIAT CLIN NEUROS | 10.1111/j.1440-1819.2007.01604.x | 226 | 12.56 | 1.21 |
| AMARE AT, 2017, TRANSL PSYCHIAT | 10.1038/tp.2016.261 | 226 | 28.25 | 5.39 |
| NIEVERGELT CM, 2006, AM J MED GENET B | 10.1002/ajmg.b.30252 | 221 | 11.63 | 0.68 |
| EDERY I, 2000, PHYSIOL GENOMICS | 10.1152/physiolgenomics.2000.3.2.59 | 217 | 8.68 | 1.76 |
| SERRETTI A, 2003, AM J MED GENET B | 10.1002/ajmg.b.20053 | 202 | 9.18 | 1.56 |
| MUKHERJEE S, 2010, BIOL PSYCHIAT | 10.1016/j.biopsych.2010.04.031 | 182 | 12.13 | 2.48 |
| MURRAY G, 2010, BIPOLAR DISORD | 10.1111/j.1399-5618.2010.00843.x | 175 | 11.67 | 2.38 |
| JONGSMA HE, 2019, LANCET PUBLIC HEALTH | 10.1016/S2468-2667(19)30056-8 | 173 | 28.83 | 4.07 |
| BENCA RM, 2008, SLEEP MED | 10.1016/S1389-9457(08)70010-8 | 163 | 9.59 | 0.84 |
| SHI JJ, 2008, AM J MED GENET B | 10.1002/ajmg.b.30714 | 156 | 9.18 | 0.8 |
| LE-NICULESCU H, 2009, AM J MED GENET B | 10.1002/ajmg.b.30887 | 155 | 9.69 | 2.36 |
| BENEDETTI F, 2005, NEUROSCI LETT | 10.1016/j.neulet.2004.11.022 | 155 | 7.75 | 1.6 |
| MANSOUR HA, 2005, CHRONOBIOL INT | 10.1081/CBI-200062413 | 155 | 7.75 | 1.6 |
| MCCARTHY MJ, 2012, J BIOL RHYTHM | 10.1177/0748730412456367 | 154 | 11.85 | 2.82 |
| FEINSTEIN A, 2004, CAN J PSYCHIAT | 10.1177/070674370404900302 | 152 | 7.24 | 1.08 |
| BENEDETTI F, 2007, AM J MED GENET B | 10.1002/ajmg.b.30475 | 149 | 8.28 | 0.8 |
| BENEDETTI F, 2004, NEUROSCI LETT-a | 10.1016/j.neulet.2003.10.021 | 142 | 6.76 | 1.01 |
| BENEDETTI F, 2004, NEUROSCI LETT | 10.1016/j.neulet.2004.06.050 | 140 | 6.67 | 0.99 |
| JAGANNATH A, 2013, CURR OPIN NEUROBIOL | 10.1016/j.conb.2013.03.008 | 135 | 11.25 | 2.17 |
| ETAIN B, 2011, EUR NEUROPSYCHOPHARM | 10.1016/j.euroneuro.2011.07.007 | 133 | 9.5 | 1.88 |
| SAUS E, 2010, HUM MOL GENET | 10.1093/hmg/ddq316 | 129 | 8.6 | 1.76 |
| SMOLENSKY MH, 2016, CHRONOBIOL INT | 10.1080/07420528.2016.1184678 | 128 | 14.22 | 3.52 |
| ROBINSON RG, 1997, ANNU REV MED | NA | 125 | 4.46 | 1 |
| MCQUILLIN A, 2007, PHARMACOGENET GENOM | 10.1097/FPC.0b013e328011b5b2 | 124 | 6.89 | 0.67 |
| EVERETT LJ, 2014, TRENDS ENDOCRIN MET | 10.1016/j.tem.2014.06.011 | 124 | 11.27 | 2.66 |
| MCCARTHY MJ, 2012, PLOS ONE | 10.1371/journal.pone.0032091 | 123 | 9.46 | 2.25 |
| LANDGRAF D, 2014, CURR PSYCHIAT REP | 10.1007/s11920-014-0483-7 | 123 | 11.18 | 2.64 |
| MANSOUR HA, 2009, BIPOLAR DISORD | 10.1111/j.1399-5618.2009.00756.x | 122 | 7.63 | 1.86 |
| MCCLUNG CA, 2011, EUR NEUROPSYCHOPHARM | 10.1016/j.euroneuro.2011.07.008 | 121 | 8.64 | 1.71 |
| GILBODY S, 2019, LANCET PSYCHIAT | 10.1016/S2215-0366(19)30047-1 | 121 | 20.17 | 2.85 |
| BUNNEY BG, 2013, BIOL PSYCHIAT | 10.1016/j.biopsych.2012.07.020 | 119 | 9.92 | 1.91 |
| MANSOUR HA, 2005, ANN MED | 10.1080/07853890510007377 | 116 | 5.8 | 1.2 |
| BENEDETTI F, 2008, NEUROSCI LETT | 10.1016/j.neulet.2008.09.002 | 116 | 6.82 | 0.59 |
| FALCÓN E, 2009, NEUROPHARMACOLOGY | 10.1016/j.neuropharm.2008.06.054 | 114 | 7.13 | 1.74 |
| LAVEBRATT C, 2010, PLOS ONE | 10.1371/journal.pone.0009407 | 113 | 7.53 | 1.54 |
| TAKAESU Y, 2018, PSYCHIAT CLIN NEUROS | 10.1111/pcn.12688 | 111 | 15.86 | 3.32 |
| LE-NICULESCU H, 2007, AM J MED GENET B | 10.1002/ajmg.b.30481 | 110 | 6.11 | 0.59 |
| CHARRIER A, 2017, INT J MOL SCI | 10.3390/ijms18050938 | 109 | 13.63 | 2.6 |
| LIN E, 2018, FRONT PSYCHIATRY | 10.3389/fpsyt.2018.00290 | 109 | 15.57 | 3.26 |

| Notes: TC: Total Citations. |
| --- |

**Table S2**. Bibliometric Indicators of High-Impact Journals.

| **Journal** | **H-index** | **G-index** | **M-index** | **IF 2023** | **JCR 2023** | **TP** | **TP-rank** | **TC** | **TC-rank** | **PY-start** |
| --- | --- | --- | --- | --- | --- | --- | --- | --- | --- | --- |
| CHRONOBIOLOGY INTERNATIONAL | 15 | 20 | 0.750 | 2.2 | Q3 | 20 | 2 | 654 | 7 | 2005 |
| JOURNAL OF AFFECTIVE DISORDERS | 15 | 23 | 1.000 | 4.9 | Q1 | 23 | 1 | 823 | 3 | 2010 |
| AMERICAN JOURNAL OF MEDICAL GENETICS PART B-NEUROPSYCHIATRIC GENETICS | 12 | 13 | 0.545 | 1.6 | Q3 | 13 | 3 | 702 | 6 | 2003 |
| BIPOLAR DISORDERS | 11 | 11 | 0.688 | 5 | Q1 | 11 | 5 | 645 | 8 | 2009 |
| MOLECULAR PSYCHIATRY | 11 | 11 | 0.647 | 9.6 | Q1 | 11 | 6 | 777 | 4 | 2008 |
| PLOS ONE | 10 | 13 | 0.667 | 2.9 | Q1 | 13 | 4 | 505 | 14 | 2010 |
| BIOLOGICAL PSYCHIATRY | 9 | 10 | 0.600 | 9.6 | Q1 | 10 | 7 | 970 | 2 | 2010 |
| NEUROSCIENCE LETTERS | 8 | 9 | 0.364 | 2.5 | Q3 | 9 | 10 | 323 | 19 | 2003 |
| LANCET PSYCHIATRY | 7 | 9 | 0.778 | 30.8 | Q1 | 9 | 9 | 41 | 122 | 2016 |
| NEUROPSYCHOPHARMACOLOGY | 7 | 8 | 0.350 | 6.6 | Q1 | 8 | 11 | 557 | 10 | 2005 |
| TRANSLATIONAL PSYCHIATRY | 7 | 7 | 0.583 | 5.8 | Q1 | 7 | 12 | 195 | 30 | 2013 |
| FRONTIERS IN PSYCHIATRY | 6 | 9 | 0.600 | 3.2 | Q2 | 9 | 8 | 60 | 88 | 2015 |
| EUROPEAN NEUROPSYCHOPHARMACOLOGY | 5 | 6 | 0.250 | 6.1 | Q1 | 6 | 13 | 207 | 26 | 2005 |
| GENES BRAIN AND BEHAVIOR | 5 | 5 | 0.294 | 2.4 | Q3 | 5 | 15 | 237 | 24 | 2008 |
| JOURNAL OF NEURAL TRANSMISSION | 5 | 5 | 0.385 | 3.2 | Q2 | 5 | 16 | 70 | 77 | 2012 |
| PSYCHIATRY RESEARCH | 5 | 6 | 0.417 | 4.2 | Q1 | 6 | 14 | 461 | 16 | 2013 |
| EUROPEAN ARCHIVES OF PSYCHIATRY AND CLINICAL NEUROSCIENCE | 4 | 4 | 0.250 | 3.5 | Q2 | 4 | 18 | 84 | 67 | 2009 |
| FRONTIERS IN BEHAVIORAL NEUROSCIENCE | 4 | 4 | 0.364 | 2.6 | Q3 | 4 | 19 | 40 | 123 | 2014 |
| JOURNAL OF BIOLOGICAL RHYTHMS | 4 | 4 | 0.267 | 2.9 | Q2 | 4 | 22 | 382 | 17 | 2010 |
| JOURNAL OF PSYCHIATRIC RESEARCH | 4 | 4 | 0.286 | 3.7 | Q1 | 4 | 23 | 169 | 37 | 2011 |

Notes: H-index: The index measures both the productivity and citation impact of the publications. IF: Impact Factor, indicating the average number of citations to recent articles published in the journal. JCR: The quartile ranking of the journal in the Journal Citation Reports, indicating the journal's ranking relative to others in the same field (Q1: top 25%, Q2: 25%-50%, Q3: 50%-75%, Q4: bottom 25%). TP: Total Publications. TP-rank: Rank of Total Publications. TC: Total Citations. TC-rank: Rank of Total Citations. Average Citations: The average number of citations per publication. PY-start: Publication Year Start, indicating the year the journal started publication.

**Table S3.** Publication and Citation Profiles of Leading Countries.

| **Country** | **Articles** | **Freq** | **SCP** | **MCP** | **MCP-Ratio** | **TP** | **TP-rank** | **TC** | **TC-rank** | **Average Citations** |
| --- | --- | --- | --- | --- | --- | --- | --- | --- | --- | --- |
| USA | 148 | 0.370 | 107 | 41 | 0.277 | 673 | 1 | 11381 | 1 | 76.9 |
| ITALY | 37 | 0.093 | 27 | 10 | 0.270 | 139 | 2 | 1998 | 2 | 54 |
| CHINA | 25 | 0.063 | 22 | 3 | 0.120 | 117 | 4 | 349 | 11 | 14 |
| JAPAN | 25 | 0.063 | 19 | 6 | 0.240 | 110 | 5 | 1107 | 4 | 44.3 |
| FRANCE | 20 | 0.050 | 13 | 7 | 0.350 | 134 | 3 | 934 | 5 | 46.7 |
| UNITED KINGDOM | 18 | 0.045 | 11 | 7 | 0.389 | 98 | 6 | 1192 | 3 | 66.2 |
| GERMANY | 17 | 0.043 | 13 | 4 | 0.235 | 71 | 8 | 403 | 9 | 23.7 |
| SOUTH KOREA | 17 | 0.043 | 11 | 6 | 0.353 | 79 | 7 | 558 | 7 | 32.8 |
| AUSTRALIA | 9 | 0.023 | 4 | 5 | 0.556 | 41 | 12 | 708 | 6 | 78.7 |
| BRAZIL | 8 | 0.020 | 4 | 4 | 0.500 | 36 | 13 | 217 | 13 | 27.1 |
| CANADA | 8 | 0.020 | 6 | 2 | 0.250 | 54 | 9 | 386 | 10 | 48.2 |
| POLAND | 8 | 0.020 | 5 | 3 | 0.375 | 20 | 18 | 202 | 15 | 25.2 |
| AUSTRIA | 7 | 0.018 | 3 | 4 | 0.571 | 24 | 14 | 211 | 14 | 30.1 |
| INDIA | 7 | 0.018 | 4 | 3 | 0.429 | 20 | 17 | 127 | 17 | 18.1 |
| SPAIN | 7 | 0.018 | 5 | 2 | 0.286 | 53 | 10 | 490 | 8 | 70 |
| SWEDEN | 6 | 0.015 | 5 | 1 | 0.167 | 24 | 16 | 346 | 12 | 57.7 |
| NORWAY | 4 | 0.010 | 2 | 2 | 0.500 | 43 | 11 | 108 | 19 | 27 |
| SWITZERLAND | 4 | 0.010 | 2 | 2 | 0.500 | 14 | 19 | 114 | 18 | 28.5 |
| FINLAND | 3 | 0.008 | 3 | 0 | 0.000 | 12 | 20 | 151 | 16 | 50.3 |
| CZECH REPUBLIC | 2 | 0.005 | 2 | 0 | 0.000 | 3 | 37 | 83 | 22 | 41.5 |

Notes: Articles: Publications of Corresponding Authors only. Freq: Frequence of Total Publications. SCP: Single Country Publications. MCP: Multiple Country Publications. MCP-Ratio: Proportion of Multiple Country Publications. TP: Total Publications. TP-rank: Rank of Total Publications. TC: Total Citations. TC-rank: Rank of Total Citations. Average Citations: The average number of citations per publication.

**Table S4**. Publication and Citation Profiles of High-Impact Authors.

| **Author** | **H-index** | **G-index** | **M-index** | **PY-start** | **TP** | **TP-Frac** | **TP-rank** | **TC** | **TC-rank** |
| --- | --- | --- | --- | --- | --- | --- | --- | --- | --- |
| MCCLUNG COLLEEN A. | 19 | 21 | 1.06 | 2007 | 21 | 6.54 | 1 | 2422 | 1 |
| MCCARTHY MICHAEL J. | 14 | 16 | 0.93 | 2010 | 16 | 4.33 | 2 | 831 | 8 |
| BENEDETTI FRANCESCO | 11 | 12 | 0.61 | 2007 | 12 | 2.27 | 3 | 513 | 10 |
| DALLASPEZIA SARA | 10 | 11 | 0.56 | 2007 | 11 | 2.19 | 4 | 500 | 11 |
| WELSH DAVID K. | 10 | 10 | 0.77 | 2012 | 10 | 2.22 | 9 | 673 | 9 |
| IWATA NAKAO | 9 | 11 | 0.53 | 2008 | 11 | 0.92 | 5 | 433 | 15 |
| KISHI TARO | 9 | 10 | 0.53 | 2008 | 10 | 0.75 | 6 | 424 | 16 |
| KITAJIMA TSUYOSHI | 9 | 10 | 0.53 | 2008 | 10 | 0.83 | 7 | 354 | 19 |
| OKOCHI TOMO | 9 | 10 | 0.53 | 2008 | 10 | 0.75 | 8 | 424 | 16 |
| OZAKI NORIO | 8 | 9 | 0.47 | 2008 | 9 | 1.23 | 12 | 345 | 20 |
| BELLIVIER FRANK | 7 | 9 | 0.50 | 2011 | 9 | NA | NA | 320 | 23 |
| ETAIN BRUNO | 7 | 9 | 0.50 | 2011 | 9 | 1.19 | 11 | 343 | 22 |
| KAWASHIMA KUNIHIRO | 7 | 8 | 0.41 | 2008 | 8 | 1.05 | 14 | 312 | 24 |
| KELSOE JOHN R | 7 | 8 | 0.47 | 2010 | 8 | NA | NA | 473 | 12 |
| COLOMBO C | 6 | 6 | 0.27 | 2003 | 6 | NA | NA | 1001 | 3 |
| DMITRZAK-WEGLARZ MONIKA | 6 | 7 | 0.55 | 2014 | 7 | 0.60 | 15 | 179 | 41 |
| INADA TOSHIYA | 6 | 7 | 0.38 | 2009 | 7 | 1.30 | 16 | 250 | 31 |
| ALDA MARTIN | 5 | 6 | 0.27 | 2003 | 6 | 0.40 | 26 | 1001 | 3 |
| FRANK ELLEN | 5 | 5 | 0.46 | 2014 | 5 | 0.46 | 27 | 171 | 42 |
| FUKUO YASUHISA | 5 | 5 | 0.31 | 2009 | 5 | 0.46 | 18 | 291 | 25 |

Notes: H-index: The index measures both the productivity and citation impact of the authors. G-index: The index gives more weight to highly cited authors. M-index: The index is the H-index divided by the number of years since the first published paper. TP: Total Publications. TP-Frac: Total Publications Fractionalized. TP-rank: Rank of Total Publications. TC: Total Citations. TC-rank: Rank of Total Citations. Average Citations: The average number of citations per publication. PY-start: Publication Year Start, indicating the year the journal started publication.
